# “Maybe I Will Just Send a Quick Text…” – An Examination of Drivers’ Distractions, Causes, and Potential Interventions

**DOI:** 10.3389/fpsyg.2017.01957

**Published:** 2017-11-17

**Authors:** Ole J. Johansson, Aslak Fyhri

**Affiliations:** Institute of Transport Economics, Oslo, Norway

**Keywords:** traffic psychology, distraction, the Big Five personality theory, the Theory of Planned Behavior, implementation intentions

## Abstract

Many people use cars all over the world. This is, however, not done without risk, as traffic accidents are one of the most common causes of death for adolescents worldwide. The number of deaths has steadily decreased, both worldwide and in Norway. Many of these accidents involve passenger cars and distracted driving. While there are many campaigns to improve safety in traffic, little research has looked at distractions. A recent report has investigated the occurrence of and damage caused by distraction, and one article has looked at what predicts baseline differences in levels of distracted driving. However, no one has tested an intervention to decrease distracted behavior in traffic. Motivational variables suggested by the Theory of Planned Behavior, personality traits, and demographic variables show utility in similar contexts and are all tested in this project. Data from two samples were collected to investigate the nature of distractions in traffic, what factors predict baseline levels of distractions, and to test an intervention to reduce distractions. Both samples feature randomly assigned intervention and control groups. The first sample (*n* = 1100 total; *n* = 208 was licensed to drive) consisted of high school students from all over Norway as a part of a larger attitudinal campaign, while the second sample (*n* = 414) was more general. The second tested a digital version of implementation intentions designed as volitional help sheets. The results from both samples suggest that there are some robust differences between people in how much they are distracted in everyday life, while some variables need further research. The second study failed to uncover any effects of the intervention. Reasons for this are discussed, along with points on the efficacy of digital interventions, the design of the volitional help sheets, and the design of the study in general. Notwithstanding the ineffectual interventions, this study contains novel information about baseline differences in distractive behavior that may further impact future behavior change interventions and guide future research.

## Introduction

Many people across the world drive cars daily. An estimate suggests that upward of 50 million people are hurt each year in road crashes, and more than one million people die (WHO, 2016). WHO suggests that people not using seatbelts or motorcycle helmets, non-adherence to speed limits and distracted driving are two main causing factors of these accidents. 2015 had the lowest number of lethal traffic accidents in Norway for more than 60 years. New technological innovations have improved safety in traffic and may continue to do so (Vaa et al., 2014). Further innovations in social science may also lead to a decrease in accidents, as interventions increase in efficacy. This article assesses the utility of an implementation intention intervention in decreasing distracted driving, and tries to examine what factors impact this utility (Gollwitzer, 1999). It also investigates drivers’ distractions generally and tests variables in predicting baseline differences in distracted behavior among two different samples.

A recent report concluded that distracted driving plays a part of at least 12% of car accidents in many different contexts and countries, with most estimates suggesting larger numbers (Sagberg and Sundfør, 2016). Many distracting factors have suggested, such as events occurring outside the vehicle, adjusting radio/cassette/CD controls, and interactions with other occupants inside the vehicle (Stutts et al., 2001). They also emphasize the rapid growth of technology, with mobile phones as a prominent example of factors that can lead to greater inattention among drivers.

Distracting factors are closely related to attention. As the task of driving a vehicle is mostly taxing on the systems of visual attention, any factor that draws the gaze away from the road for a significant period of time could be classified as a distractor (Sagberg et al., 2016). Specifically, after 2 s of distraction, the risk of getting involved in an accident increases drastically (Sagberg and Sundfør, 2016). Attention has been conceptualized as an array of systems that select to focus on some sensory stimuli while discarding others (Reisberg, 2013). The systems of attention can be categorized into top-down and bottom-up systems which work in different ways while driving (Petersen and Posner, 2012). The former is attention guided by volition, a proactive and cognitively adjusted way of controlling what to focus on, such as making a phone call whilst driving. The bottom-up approach is a reactive, stimulus-driven approach, and is relevant when stimuli draw our attention without our conscious control such as receiving a phone call. A better understanding of how to effectively deal with stimuli that fight over this limited resource could potentially reduce the number of accidents on roads. However, predicting and explaining human behavior is difficult, especially if people act in discordance with their own intentions.

In the 1980’s, the Theory of Planned Behavior (TPB) was introduced as an improved framework for explaining human behavior (Vaughan and Hogg, 2005). Building on the Theory of Reasoned Action (TRA), it included a measure of perceived behavioral control (PBC), in addition to attitudes toward a behavior and perceived social norms. These variables are thought to predict intentions to perform a range of behaviors (Ajzen, 1985). Intention would then be highly associated with performing the behavior in question, mediating all the variance between the motivators and behavior. One exception has been made with PBC, as that sometimes can lead directly to performing the behavior in question. While this approach has proven itself as a good way of explaining human variance in both intentions and behavior, intentions may predict too little of the behavioral variance in some domains (Ogden, 2012), and several meta-analytic reviews have found the efficacy of the model to vary between behaviors and studies (Ajzen, 1991; Godin and Kok, 1996; McEachan et al., 2011). Estimates place the explained variance in intentions as generally between 40 and 50%, and the explained variance in behavior between 19 and 38% (Ogden, 2012). No research has investigated the theory’s efficacy in relation to drivers’ distractions in traffic. Those with intentions to reach a goal, but who fail to do so, have been labeled *inclined abstainers.* These people have been found primarily to be responsible for the intention–behavior gap found in the TPB (Gollwitzer and Sheeran, 2006). Research into the intention–behavior discrepancies found different problems leading to an intention–behavior gap (Prestwich et al., 2015). These concerned opportunities to express intentions into behavior, changing saliency of cues, and lack of elaboration of cues to action. These three problems have been addressed with recent interventions.

In the late 1990’s, Gollwitzer’s paper titled “*Strong effects of simple plans*” lay the foundation for what has become a recognized psychological intervention (Gollwitzer, 1999). Implementation intentions are designed specifically to address the apparent gap between intention and behavior, and to close it as far as possible (Gollwitzer, 1999). It does this by making people form plans following an “if-then”-structure to reach the goals they set themselves. Personal goals, persistence with boring tasks, increased performance in dichotic listening tasks and returning postcards have all benefited from the kind of planning involved in implementation intentions (Gollwitzer and Sheeran, 2006). Effect sizes for intervention studies using an implementation intention design have been approximately medium (Prestwich et al., 2015). For emotional control, a high effect size of *d* = 0.91 was observed (Webb et al., 2012), and a low one was noted for physical activity *d* = 0.30 (Carraro and Gaudreau, 2013). While mostly used for health behaviors, it has also proven effective in general applications (Gollwitzer and Sheeran, 2006; Prestwich et al., 2015). Especially important to the plan’s efficacy, is participants’ baseline intentions to perform a behavior.

Participants in studies using implementation intentions are usually told to make plans for a specific aspect of their lives they want to change (Sheeran, 2002). Planning using an implementation intention design, involves semantically combining the when, where, and how of achieving that goal. For example, a person may combine the “if”-statement: “if I am tempted to drive faster than the speed limit while on the highway…” with the “then”-statement: “then I will remind myself that it is dangerous and illegal to do so.” These if-then planning interventions usually are accompanied by careful instructions, as the quality of the plan has a large impact on the efficacy of the intervention (de Vet et al., 2011a).

Research suggests that more than 30% do not make plans when asked to, and that roughly 30% of those who do plan, fail to make good plans (Michie and Abraham, 2004; de Vet et al., 2011b). Their plans are often too general and lack specification of behavioral cues and appropriate actions (de Vet et al., 2011a). A high-quality plan for physical exercise would work to defeat habit, not only by specifying that the workout should be done in the evening, but also which evening and at what time. The habitual nature of many car driving tasks, such driving too fast or adjusting the radio, is just what the implementation intentions aim to alter (Gollwitzer, 1999). The process of making the plans is thought to fortify and complete the planning effects, and properly elaborating on the possible situations and cues is crucial for planning with good effect (Prestwich et al., 2015).

To mitigate poor planning, some research groups have tried to standardize the induction of implementation intentions through *volitional help sheets* (Arden and Armitage, 2012; Armitage, 2015; Brewster et al., 2015). While most people can form self-regulatory strategies themselves, standardizing them leads to greater experimental control, and eases the process of intervention (Brewster et al., 2015). It has also been argued that people often fail to make high-quality plans, or fail to follow the format at all (Michie and Abraham, 2004; de Vet et al., 2011a). Volitional help sheets list pre-made critical situations and behavioral solutions, and tell participants to link situations and strategies. This method has proven successful in some studies, with no decrease in the efficacy of the intervention. The list of critical situations in which they are likely to not act according to their goals constitutes the “if”-part. These situations are often empirically derived, but can also be synthesized from theoretical frameworks. After choosing critical situations, they are asked to link these, typically by drawing a line, with appropriate coping strategies or “then”-statements. As stated in the original paper, successful goal striving often relies on finding purposeful, instrumental behaviors that bring people closer to their goals (Gollwitzer, 1999). It is not clear that strategies based on some theories are superior in achieving behavior change than other theories or basing them on empirical data of what people already do to achieve their goals.

There is little doubt that there are systematic differences between groups of people in how they behave in traffic and how they respond to planning interventions. For example, there exists considerable gender and age differences in who ends up in traffic accidents, with young males being most accident prone (Statistisk Sentralbyrå, 2016; Vegdirektoratet, 2017). Young male drivers are more neurologically predisposed to sensation-seeking and risky behaviors (Arnett, 1996; Byrnes et al., 1999). In addition, young drivers will often lack experience and get in more accidents due to risky behavior (Turner and McClure, 2003; Rundmo and Iversen, 2004). In opposition, older drivers are found to be more inattentive drivers (Aberg and Rimmo, 1998), and some have even found that females are more prone to being distracted (Bone and Mowen, 2006). These contradictions further the importance of more knowledge about demographic information and distractions. For example, how often one drives is suggested as an important predictor of distractions as those who drive more can habituate to the task and grow bored of it, thus seeking other stimulants (Bone and Mowen, 2006). Similar situations elicit different responses from people, and this is a classic area of application for taxonomies of personality psychology (Holt et al., 2012).

The Big Five is one of the most prominent and praised personality taxonomies (McCrae and Allik, 2002). The model describes the tendencies people have to act and think in certain ways using only five bi-dimensional traits on which people score high or low; namely extraversion (E), neuroticism (N), openness to experience (O), conscientiousness (C), and agreeableness (A). It has been found to predict traffic behavior generally, and explain dangerous behavior specifically (Salgado, 2002; Sümer et al., 2005). Short-form measures of the Big Five variables have been developed to make it more accessible and easy to use in diverse domains (Donnellan et al., 2006). Some researchers have found that personality factors impact safe driving, but not distracted driving in particular (Jiang et al., 2011). Conscientiousness has specifically been shown to predict risky driving, along with some personality facets associated with extraversion (Schwebel et al., 2006). In fact, some have found conscientiousness to be a prominent predictor of total accidents (Arthur and Doverspike, 2001). One study showed how all the different aspects of the Big Five explain accident risk through aberrant driving behavior, suggesting that personality may be mediated by other variables (Sümer et al., 2005). The other Big Five-variables also seem to explain some of the interpersonal variance in accident proneness, which is suggestive of a complex interplay and an intricate causal model.

When looking at distractions, and especially a planning intervention to reduce it, conscientiousness, extraversion, and neuroticism may play more of a role than the others. Conscientiousness has generally been the more effective as it has shown predictions of distracted driving, and may interact with a planning intervention (Arthur and Doverspike, 2001; Sümer et al., 2005; Bone and Mowen, 2006; Webb et al., 2007; Ajzen et al., 2009). Other research also indirectly support both a main and interactional effect of conscientiousness in health-related behavior (Bogg and Roberts, 2004). Extraversion closely relates to sensation-seeking or boredom while driving, which could be specially relevant for younger drivers (Arnett, 1996; Rundmo and Iversen, 2004). Neuroticism could be included because it relates to reactive behaviors that could be of importance in drivers’ distractions (Jovanović et al., 2011; Thørrisen, 2013). Furthermore, an increase in neuroticism may increase baseline levels of anxiousness or tenseness, which are hypothesized to worsen the impact of negative events on driving performance (ibid.).

The present article aims to explain and reduce drivers’ distractions by testing variables suggested by the TPB, personality, demographics, and the efficacy of an implementation intentions intervention. Specifically, the article aims to test the following hypotheses.

*The first hypothesis (H1)* is that both driving more often and longer will positively predict distracted behavior.
*The second hypothesis (H2)* is that motivational pre-cursors of behavior suggested by the TPB will predict distractive behavior at baseline. More lenient norms and attitudes will increase level of distractions, and a high PBC for avoiding distracted driving will yield less distracted behavior. PBC for general driving will also be a positive predictor.
*The third hypothesis (H3)* is that traits of personality will predict levels of distractive behavior at baseline. Specifically, we expect neuroticism and extraversion to positively predict, and conscientiousness to negatively predict drivers’ distractions.
*The fourth hypothesis (H4)* is that forming implementation intentions using an online version of volitional help sheets will reduce drivers’ distractions.
*The fifth hypothesis (H5)* is that the effect of forming implementation intentions will be stronger for the inclined abstainers in the sample.
*The sixth hypothesis (H6)* is that conscientiousness will moderate the effect of the planning intervention on top of intentions.

In addition, the data will be explored using correlation and descriptive statistics, especially regarding the nature of distractive behavior. Age and gender will also be explored for effects on baseline levels of distractions as the directionality of the effect is unclear. Further exploration will be done regarding the volitional help sheet, whether theoretically derived solutions from the stages of change-model, or empirical solutions derived from recent reports and a pilot study seem more efficacious.

## Materials and Methods

### Sampling

Two separate samples were collected to test the hypotheses. The first consisted of high school students (*n* = 1100) and focused on baseline measures and describing distractions among youth in Norway. The second sample consisted of a random sample of Norwegians (*n* = 617), and tested both baseline predictions and the intervention, using two experimental groups and an active control group. Both data collections were approved by Norwegian Centre for Research Data (NSD).

Participants in the first sample were recruited through the traffic safety organization Trygg Trafikk and their county departments. All participating schools were chosen randomly and represented seven different counties in Norway, while the second sample was a representative, randomized Norwegian sample who had been recruited during a previous study by the Institute of Transport Economics (TØI). In both instances, a small gift certificate was promised to a couple of lucky participants. For the first sample, contact with schools recruited by Trygg Trafikk was upheld by their county departments. Instruction on how to best complete the survey followed the link in an email. The pre-survey was administered in the first 2 weeks of October 2016. The second sample was contacted directly using emails in March 2017. The second survey was designed as a replication of and improvement over the first; scales’ wording were reviewed to improve psychometric properties, and demographic variables lacking efficacy in regression models were removed or redesigned (see below). The TPB and Big Five scales were increased from five- to seven-point to increase response variance (Bordens and Abbott, 2011, pp. 265–266). All scales showed acceptable internal consistency for longer scales, and strong correlations between items for shorter ones (DeVellis, 2003).

### Demographics

The questionnaire was designed to measure drivers’ distractions and general individual difference information. Both samples’ surveys featured demographic information, such as how large their home town was, age, gender, and information about transportation habits and completed traffic education. For the first sample, information specific to high school was included, and was substituted for information about education levels in the second.

### The Big Five

For both samples, short-versions of the three selected traits were used. These consists of four-item scales which were chosen for their utility and effectivity (Donnellan et al., 2006). The items were translated and wordings was checked against previous translations of Big Five measures for extraversion, neuroticism, and conscientiousness (Engvik and Føllesdal, 2005).

### Theory of Planned Behavior

The motivational pre-cursors subjective norms, attitudes, PBC, and intentions were also measured using composite scales in regard to driver distractions in both samples. Previous Norwegian research was influential in designing the questions (Moan, 2005). We used two items to measure normative beliefs, five bi-dimensional axes to measure attitudes, and seven items for PBC for distracted driving and two for general driving. Due to technical error, intention was only measured for the second sample using three items. Intention was also designed to control for inclined abstainers, by asking people the degree to which they wanted to be safer drivers.

### Distracted Behavior

Last, the survey included 11 items measuring drivers’ distractions during the last 2 weeks. These 11 items were informed by recent reports and a pilot study (Sagberg and Sundfør, 2016). They were grouped into two groups of distractions: mobile phone use and secondary tasks. These were thought to be often occurring among the target population and suitable for intervention. Participants rated how often these distracting behaviors occurred on a six-point scale from 1 “Never” to 6 “Very often.” The two categories were also combined into a general distraction index. The second sample also got asked about their perception of the relevance of the plans they had made. Also included here was a measure of how often they performed behaviors, without mention of distractions. The thought was to investigate whether people did the behaviors without getting distracted. An open comment section was also implemented.

### Implementation Intention Intervention

The second sample received an intervention based on implementation intentions. It was put at the end of the T0 measures, and featured a digital volitional help sheet (see Supplementary Figures 1–4). The critical situations in this sheet closely resembled baseline measures of distracted behavior. As an area of exploration, half of the solutions for mobile phone distractors were empirically derived from previous research, while the other half was theoretically derived, and focused on the stages of change model (Prochaska et al., 1988; Brewster et al., 2015). A digital design for delivering the volitional help sheet version of the implementation intention intervention was developed to make the plan formation as engaging, yet easy, as possible (see Supplementary Material). First, each participant was presented with a list of pre-defined distracting situations at the end of the survey. They were told to choose relevant ones, and thus complement them with pre-defined behavioral solutions. Every participant made two plans for mobile phone use, two for secondary distractions, and one for when they get in their cars. To maximize intervention efficacy, reminders of participants’ plans were sent out 1 week after induction (Prestwich et al., 2009). The control group was presented with standardized information about distractions in traffic and asked to rank a list of distractors by their disruptiveness to traffic safety. Respondents were grouped by the date of their completion of the baseline survey, so that the follow-up survey would arrive close to 2 weeks after their completion of the first.

## Results

Data analysis for testing hypotheses in both samples consists of three general procedures: correlation, regression, and ANOVA. For hypotheses one, two, and three, correlations provided the first step toward information about the interplay between the variables in this article. For a more conclusive test of these three hypotheses a multiple regression was used. In the second sample, ANOVAs were used to test randomization and drop-out. To test hypotheses four, five, and six, a repeated measures ANCOVA was used. Here, different analyses of variance were utilized to explore the data and uncover other trends.

### Descriptive Results from the Student Sample

There were 1,100 respondents in the first sample with a mean age of 17.2 (*SD* = 1.63). The range in overall reported age was from 15 to 30, and there was a slight gender skew overall (42.6% men, 57.4% women). There was an equal split between those who lived in towns with more than 10.000 inhabitants (40.1%) and those who lived in towns with less (43.3%), suggesting an even rural/urban representation in the sample. Some reported driving cars as their most common means of transportation (14.9%), while most people reported mostly being passengers (40.8%). Two hundred and eight students (18.9%) reported having the drivers’ license. Most of the respondents reported having their license for less than 6 months (53.6%) at T0, while 10% reported more than 25 months. Most drivers (53.9%) reported driving more than 10 times the last 2 weeks and most students drove cars more than 40 km the last 2 weeks (62.4%).

In **Table 1**, main variables and their descriptive statistics are shown. It is worth noting the differences in number of included students, as only 18.9% of the total sample were licensed to drive. In order to favor statistical power, and to better describe a younger population as a whole, all respondents are included where applicable.

**Table 1:** Descriptive statistics for main variables in the first sample.

	*N*	Mean	*SD*	Skewness	Kurtosis
Age	1,100	17.2	1.63	2.86	14.88
Often driven^a^	208	4.40	1.51	–0.61	–0.66
Far driven^a^	208	4.03	1.69	–0.34	0.11
Attitude^b^	1,100	2.44	0.59	0.12	–1.11
Social norms^b^	1,100	4.02	0.79	–0.61	0.11
PBC^b^	1,100	3.57	0.54	0.01	0.26
PBC driving^b^	207	4.02	0.70	–1.30	0.02
Extroversion^b^	1,100	3.21	0.91	–0.20	3.90
Neuroticism^b^	1,100	2.72	0.83	0.13	–0.46
Conscientiousness^b^	1,100	3.62	0.71	–0.44	–0.33
Distractive behavior^a^	207	2.39	1.11	0.85	0.04

*^a^Six-point scales, ^b^Five-point scales.*

All three personality constructs show mean scores revolving around the semantic mean of three, with conscientiousness slightly higher. When looking only at those who already had a driver’s license, the mean age went up to 19.0 (*SD* = 2.06), and the gender skew shifts toward more males (58.2%). A somewhat low mean for attitudes suggests that there generally is a negative perception of driving while distracted and a high mean for norms suggest that respondents think their peers dislike when they let themselves get distracted. It is also apparent that our sample finds it neither hard nor easy to avoid being distracted while driving, with a PBC mean closer to the middle of the scale. They also find it quite easy to drive, with a mean close to the high-end of the scale.

Overall, the students reported low levels of driver distraction. Although, as shown in **Table 2**, some distractions occur more often than others. Their distribution seem consistent and somewhat large, suggesting some difference between participants. Item 1, “Operating the radio” has the highest score with people on average being distracted by it between “rarely” and “sometimes.” Item 9, “Writing a message on the phone,” seems to be the least occurring distraction in my pre-survey sample and people on average get distracted by this less than “very rarely.”

**Table 2:** Means of each distractive behavior from the first sample (*n* = 206).

Items		Mean	*SD*
1	Operating the radio	3.58	1.50
2	Handling navigational devices	2.15	1.34
3	Handling equipment in the car	2.44	1.35
4	Eating or drinking	2.62	1.48
5	Prolonged eye-contact with passenger	2.38	1.31
6	Reaching for an object in the car	2.41	1.35
7	Answering incoming calls	2.41	1.54
8	Making calls	2.18	1.48
9	Writing a message	1.93	1.30
10	Reading a message	2.10	1.35
11	Other use	2.28	1.37
	Total	2.39	1.11

### Testing Baseline Differences in the Student Sample (H1, H2, H3)

A correlation matrix was used as a first step toward testing hypotheses one, two, and three. The correlation matrix in **Table 3** suggests that gender, how often driven, neuroticism, and TPB-measures show significant relations with distractive behavior. It seems that women report being less distracted, and that how often respondents reported driving had significant positive relations with distractive behavior. Furthermore, a negative relationship exists between norm and gender, meaning that women perceive more negative social norms toward distracted driving. A neurotic person, but not extraverted nor conscientious, seem to report more distractions.

**Table 3:** Correlation matrix between key variables in the first sample (*n* = 1100).

		1	2	3	4	5	6	7	8	9	10	11	12	13
1	Distractive behavior^a^	–												
2	Age	0.01	–											
3	Gender^b^	–0.14^*^	0.01	–										
4	Inhabitants	0.03	–0.07^*^	0.00	–									
5	Often driven^a^	0.25^*^**	0.04	–0.05	0.03	–								
6	Far driven^a^	0.13	0.06	–0.11	–0.03	0.55^*^**	–							
7	Attitude	0.34^*^**	0.05	–0.23^*^**	0.04	0.03	0.00	–						
8	Social norms	–0.27^*^**	–0.07^*^	0.11^*^**	–0.02	0.04	0.10	–0.33^*^**	–					
9	PBC	–0.41^*^**	0.01	–0.11^*^*	–0.01	0.02	0.08	–0.03	0.16^*^**	–				
10	PBC driving^a^	–0.10	0.07	–0.14	0.08	0.20^*^*	0.14	0.11	0.02	0.41^*^**	–			
11	Extraversion	0.13	0.08	–0.06	0.05	–0.04	–0.03	0.06	–0.00	0.01	0.03	–		
12	Neuroticism	0.17^*^	–0.03	0.14^*^**	0.00	–0.05	–0.05	–0.04	0.02	–0.09^*^*	–0.23^*^*	0.17^*^**	–	
13	Conscientiousness	–0.11	0.00	0.16^*^**	–0.03	–0.02	–0.05	–0.06	0.07^*^	–0.10	0.10^*^*	0.10^*^*	0.17^*^**	–
14	Intention^c^	0.52	0.52	0.16^*^*^†^	–0.52	0.26	–0.74	0.70	–0.52	–0.76	0.09	0.57	–0.30	0.90

*^a^*n* = 207. ^b^0 = male, 1 = female. ^c^*n* = 4. ^∗^*p* < 0.05, ^∗∗^*p* < 0.01, ^∗∗∗^*p* <0.001, ^†^*n* = 400.*

A regression provided a further test of hypotheses one, two, and three for the first sample (see **Table 4**). Driving more often, having more positive views about driving while distracted, perceiving attitudes of significant others as more lenient, and perceiving driving without getting distracted as less under their control are all factors that contribute significantly to increasing distracted behavior. This partially confirms these hypotheses. While correlation did not suggest it, extraversion positively predicts self-reported distractions, as did neuroticism. Overall the explained variance in the models is high, with roughly equal amount for all three categories of distraction. Data exploration suggests that gender has a significant impact on all measures of distracted behavior, proposing that females are less distracted than males.

**Table 4:** Regression model predicting three groups of distractive behavior in the first sample (*n* = 206).

Independent	β for general	β for mobile	β for secondary
variables	distractions	phones	tasks
*R*^2^ for models	0.42	0.40	0.38
Age	–0.04	0.00	–0.07
Gender^a^	–0.17^*^*	–0.18^*^*	–0.13^*^
Often driven	0.21^*^*	0.21^*^*	0.18^*^
Far driven	0.08	0.06	0.08
Attitude	0.25^*^**	0.31^*^**	0.15^*^
Social norms	–0.15^*^	–0.10	–0.19^*^*
PBC	–0.36^*^**	–0.31^*^**	–0.37^*^**
PBC driving	–0.01	–0.04	0.03
Extraversion	0.15^*^	0.16^*^*	0.11
Neuroticism	0.25^*^**	0.21^*^*	0.27^*^**
Conscientiousness	0.02	0.05	–0.03

*^a^0 = male, 1 = female. ^∗^*p* < 0.05, ^∗∗^*p* < 0.01, ^∗∗∗^*p* < 0.001.*

### Drop-Out and Exclusion in the General Sample

Only respondents who were licensed to drive and who had driven the past 2 weeks were included in the analysis of intervention effects. Participants who had not driven the last 2 weeks at T1 were also excluded. In order to better control participant flow, only those answering within the first 2 weeks of survey distribution were included. Four hundred and fourteen participants in total were subjected to analysis after completing both data collections and meeting inclusion criteria. Six hundred and seventeen respondents were subjected to baseline analyses.

### Descriptive Results from the General Sample

Of the 1,763 emails sent, 701 (39.7%) responded at T0, with 617 remaining after exclusion. At T1, 414 of these participants were uniquely identified and carried forward for analysis of intervention effects. There was an even split between male (51.7%) and female (48.3%) respondents in this final sample. Only 4.8% of this sample reported having completed middle school as their highest level of education, with 31.6% having completed high school. Further, 29.2% reported having completed three or more years of higher education, with approximately a third (34.3%) having completed four or more years of higher education. Key statistics for variables at T0 and T1 are presented in **Table 5**.

**Table 5:** Descriptive statistics across time points for main variables in the second sample.

	T0 (*n* = 617)		T1 (*n* = 414)	
	Mean	*SD*	Mean	*SD*
Age	44.94	14.17	46.48	14.60
Often driven^a^	3.86	1.24	3.83	1.22
Far driven^a^	4.59	1.51	4.60	1.53
Attitude^b^	4.76	1.47	4.78	1.45
Social norms^c^	3.90	0.86	3.87	0.88
PBC^b^	4.77	0.99	4.78	1.00
PBC driving^c^	4.32	0.55	4.32	0.53
Extroversion^b^	3.99	1.34	3.86	1.33
Neuroticism^b^	3.10	1.28	3.14	1.31
Conscientiousness^b^	5.19	1.21	5.14	1.27
Intention to change^b^	6.44	0.85	6.42	0.88
Distractive behavior^a^	1.97	0.70	2.00	0.70

*^a^Six-point scales, ^b^Seven-point scales,^c^Five-point scales.*

It is worth noting that some numbers in **Table 5** are not directly comparable with scores from the first sample, as scales measuring personality and TPB were shifted from five-point to seven-point scales. Personality constructs suggest that this sample is higher on conscientiousness, and about middle on extroversion and neuroticism. With those who had not driven the past 2 weeks discarded, a majority of the remaining participants (56.0%) had driven more than once every day on average. Only 11.5% had driven less than 20 km during these 2 weeks, which suggests that most of those included had driven substantial distances.

The mean for attitude, PBC for distractions and PBC for driving, all score somewhat above the semantic mean. This suggests that respondents have somewhat unfavorable views about being distracted while driving, and think that they are in control both in terms of driving, and avoiding distractions while driving. Social norms, also above the semantic middle-point, suggests that respondents think their significant others generally would not like if respondents became distracted while driving. A high intention to be less distracted is encouraging, as this suggests that most respondents, while reporting being somewhat distracted, also want to be as safe drivers as possible. The three items measuring intentions to be as safe drivers as possible were compiled into a mean score. As shown in **Table 5**, there was a very high intention score in this sample. This suggests that the sample has a focus on being safe in traffic.

As in the previous sample, most self-reported distractive behavior shows relatively low frequency as displayed in **Table 6**. Most single behaviors vary around a score of two across participants, which equals “Very rarely.” The total average also suggests this, and the standard deviation shows that most respondents answer quite close to this low frequency. Consistent with the first sample, “Operating the radio” still is suggested the most frequent distractor at both T0 and T1, scoring more than one point above the lowest, “Writing a message on the phone” at T0.

**Table 6:** Means of each distractive behavior at T0 and T1 in the second sample (*n* = 414).

Items		Baseline		Follow-up	
		Mean	*SD*	Mean	*SD*
1	Operating the radio	2.60	1.17	2.12	1.10
2	Handling navigational devices	1.84	1.02	1.55	0.88
3	Handling equipment in the car	1.82	0.88	1.63	0.81
4	Eating or drinking	2.05	1.05	1.88	0.99
5	Prolonged eye-contact with passenger	2.18	0.99	1.94	0.93
6	Reaching for an object in the car	2.23	0.99	2.05	0.97
7	Answering incoming calls	2.19	1.11	2.05	1.08
8	Making calls	1.95	1.13	1.80	1.08
9	Writing a message	1.42	0.88	1.29	0.72
10	Reading a message	1.63	0.95	1.46	0.85
11	Other use	1.71	0.96	1.49	0.85
	Total	2.00	0.70	1.74	0.63

### Testing Baseline Differences in the General Sample (H1, H2, H3)

A correlation matrix from the T0 data is presented in **Table 7**, and provides a first step in exploring relationships between variables, and testing hypotheses one, two, and three. Here, we see that several variables are suggested to covariate with distracted behavior. Older, female participants are less distracted. How often and how far one drives are both linked to increased distractive behavior, while PBC for distractive behavior shares a negative relationship. More neurotic and less conscientious respondents report more distractions, and higher intentions to be a safe driver go along with fewer distractions. Furthermore, the matrix unveils several relationships between individual difference variables. Multiple gender differences were found, for example in how often participants drive, their attitudes, their neurotic tendencies and their intentions to be safe drivers. Multiple correlations also exist between TPB-variables and personality items informed by the Big Five.

**Table 7:** Correlation matrix between key variables in the second sample *(n* = 617).

		1	2	3	4	5	6	7	8	9	10	11	12	13
1	Distractive behavior	–												
2	Age	–0.28^*^**	–											
3	Gender^a^	–0.10^*^	–0.32^*^**	–										
4	Education	0.01	0.05	–0.05	–									
5	Often driven	0.28^*^**	–0.08	–0.18^*^**	–0.03	–								
6	Far driven	0.11^*^	0.12^*^	–0.32^*^**	0.03	0.48^*^**	–							
7	Attitude	–0.03	–0.24^*^**	0.11^*^	0.10^*^	–0.08	–0.10^*^	–						
8	Social norms	0.05	–0.25^*^**	0.11^*^	0.01	0.06	0.05	0.43^*^**	–					
9	PBC	–0.31^*^**	0.18^*^**	0.03	–0.06	–0.05	–0.05	–0.21^*^**	–0.11^*^	–				
10	PBC driving	0.03	0.06	–0.18^*^**	–0.03	0.14^*^*	0.12^*^	–0.17^*^*	–0.03	0.31^*^**	–			
11	Extraversion	0.07	0.04	–0.01	0.06	0.10^*^	–0.01	–0.02	0.04	–	0.11^*^	0.06	–	
12	Neuroticism	0.10^*^	–0.22^*^**	0.21^*^**	–0.14^*^*	–0.03	–0.18^*^**	0.10	0.03	–0.11^*^	–0.18^*^**	–0.14^*^*	–	
13	Conscientiousness	–0.10^*^	0.12^*^	0.02	–0.07	0.03	–0.01	–0.10^*^	–0.12^*^	0.17^*^*	0.13^*^*	0.08	–0.14^*^*	–
14	Intention	–0.27^*^**	–0.02	0.20^*^**	–0.09	–0.14^*^*	–0.08	0.19^*^**	0.21^*^**	0.15^*^*	–0.04	–0.07	0.01	0.05

*^a^0 = male, 1 = female. ^∗^*p* < 0.05, ^∗∗^*p* < 0.01, ^∗∗∗^*p* < 0.001.*

A multiple regression analyses was run to explore the data, and test hypotheses one, two, and three in the general second sample. These results are presented in **Table 8**. Education and intention to change are new variables since the first sample. Those who drive more often, but not farther, are as in the previous sample, prone to being more distracted, partially confirming hypothesis one. As the first sample’s analyses were run without the scale for intentions, we also ran a regression model without intentions for the second sample, because intentions should mediate the effects of attitudes, norms and PBC. The results showed that the regression weights of some variables, in particular of attitudes and PBC, increased in the model without intentions. However, none of the variables’ significance levels were affected by whether intentions were included or excluded. A higher score on attitudes in this second sample means a more negative view of distracted driving (unlike in the first sample, where it meant a more lenient view), attitudes negatively and significantly predict general and mobile distractions. Having a higher PBC for not being distracted significantly predicts fewer distractions for all categories as in the first sample. Having intentions to be safe drivers predicts fewer distractions for general and mobile categories, but not for secondary tasks. Different to the first sample, attitudes are not significant for secondary tasks, and social norms fail to predict level of any distracted behavior. None of the personality variables turn out significant. This confirms hypothesis one, but only partially confirms hypothesis two, and rejects hypothesis three. Age and gender show significant impacts on behavior, suggesting that young, male drivers are more distracted than their senior and female counterparts.

**Table 8:** Regression model predicting distractive behavior at T0 in the second sample (*n* = 414).

Independent	β for general	β for mobile	β for secondary
variables	distractions	phone	tasks
*R*^2^ for models	0.17	0.30	0.17
Age	–0.26^*^**	–0.27^*^**	–0.19^*^**
Gender	–0.11^*^	–0.13^*^*	–0.07
Education	0.01	–0.00	0.01
Often driven	0.19^*^**	0.22^*^**	0.12^*^
Far driven	–0.02	–0.01	–0.03
Attitude	–0.10^*^	–0.12^*^	–0.06
Social norms	0.04	0.07	–0.00
PBC	–0.26^*^**	–0.22^*^**	–0.25^*^**
PBC driving	0.07	0.08	0.04
Extraversion	0.09	0.08	0.08
Neuroticism	0.07	0.06	0.06
Conscientiousness	–0.03	–0.00	–0.06
Intention	–0.16^*^**	–0.19^*^**	–0.10

*^∗^*p* < 0.05, ^∗∗^*p* < 0.01, ^∗∗∗^*p* < 0.001.*

To test and explore the mediating effect of intentions between attitudes, norms, PBC, and distractive behavior, we ran a separate model predicting intentions. Here, attitudes (β = 0.15, *p* = 0.006), norms (β = 0.16, *p* = 0.002), and PBC (β = 0.18, *p* < 0.001) all predicted intentions while controlling for the other variables from **Table 8**. According to the four steps of mediation, the data suggest that the effect of attitudes and PBC on behavior is partially mediated by intentions (Baron and Kenny, 1986).

### Planning Efficacy

#### Tests of Attrition and Randomization

An ANOVA was conducted to test for significant differences in baseline measures between those who completed both data collections and those who dropped out (*n* = 198). The dependent variables were the baseline measures of mobile phone and secondary tasks distractions, as well as intention and motivational pre-cursors. The independent variables were whether or not they dropped out. No multivariate or between-subject main effects emerged, suggesting no difference between those who dropped out and those who did not. A similar ANOVA was run to test for difference between conditions, but no such effect was uncovered. This suggests that the randomization was successful. One further analysis was run to test for differential attrition. That is whether the drop-out rate was different for the three conditions in the sample. A Pearson’s chi-square test of independence suggested that there was no systematic difference between the cells in a crosstab [*X*^2^(2,617) = 1.56, *p* = 0.458]. Therefore, we concluded that the drop-out constituted data missing at random, and that further tests to investigate type of missingness would be unwarranted as that is difficult to uncover (Jansen et al., 2006).

#### Effects of the Planning Intervention (H4, H5, H6)

**FIGURE 1:**
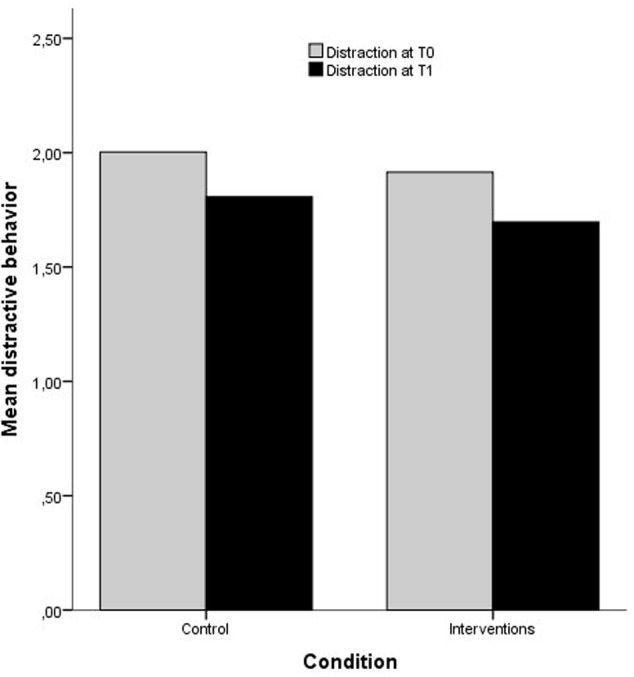
Means of distractive behavior at T0 and T1 between conditions in the second sample.

The overall means between conditions are portrayed in **Figure 1**. Here it seems that both intervention groups and the control group have a marked decline in distractive behavior. If the intervention had an effect, this decline should be greater for the intervention group. Hypothesis four, five, and six was investigated using a repeated measure ANCOVA with intentions and conscientiousness as covariates. Here, a single mean for both categories of distractive behavior was used as dependent variable, with time and condition as independent variables. No significant interaction effect between the within-subject factor *time* and the between-subject factor *condition* was found, Wilks’ λ= 1, *F*(1,412) = 0.19, *p* = 0.661, η^2^ = 0.000. This suggests that the change over time in distracted behavior is equal in the control group and intervention groups. The main effect of time proved significant with a large effect size, Wilks’ λ= 0.864, *F*(1,412) = 64.6, *p* < 0.001, η^2^ = 0.136, *d* = 0.79, which suggests that there was a marked decline in distraction for my sample in total from T0 to T1 (Cohen, 1988). This also meant that there was no effect of the included covariates.

Further repeated measure ANCOVAs were done to explore if the effect was unique to those high in intentions to be safe drivers, instead of just controlling for intentions. There was, however, no significant effect on the interaction between time, condition, and intention, Wilks’ λ= 0.959, *F*(10,389) = 1.66, *p* = 0.088, η^2^ = 0.041. The interaction between time and intention did, however, turn out significant with a medium effect size, Wilks’ λ= 0.929, *F*(13,389) = 2.30, *p* = 0.006, η^2^ = 0.071. This suggested that the decline in general distractions was greater for those with higher intentions. One further ANCOVA explored the effect of intervention on general distractions only for those who answered that the plans they made were relevant. Those answering on average above the semantic mean of 3.5 for those items were included in the analysis. The effect may have been masked by those who did not feel that the planning sections suited them personally. However, still no effect was found for the interaction of time and condition.

A similar repeated measure ANCOVA was run to explore the difference in distractions between the two kinds of behavioral strategies for mobile phone use. Here, mobile phone distraction means were used as a dependent variable, and we looked for interaction effects between either of the two dummy-coded conditions and time passed. Neither empirically derived, Wilks’ λ= 1, *F*(1,408) = 0.07, *p* = 0.792, η^2^ = 0.000, nor theoretically derived, Wilks’ λ= 0.999, *F*(1,408) = 0.23, *p* = 0.633, η^2^ = 0.001, turned out significant. This suggested that neither kinds of intervention were able to change mobile phone distractions.

An additional repeated measures ANCOVA was run to test and explore the differences in effect on secondary tasks and mobile phone use. No significant effect of intervention was found. Not for secondary distractions’ interaction with condition, Wilks’ λ= 0.999, *F*(1,409) = 0.44, *p* = 0.508, η^2^ = 0.001, nor for mobile phone uses’ interaction with condition, Wilks’ λ= 1, *F*(1,409) = 0.16, *p* = 0.689, η^2^ = 0.000.

#### Testing Floor Effects

For the respondents that reported being very little distracted at the onset, their possible behavior intervention gain was very low. Thus, we wanted to explore if there only was an effect for respondents who reported above mean levels of distractive behavior. However, when running a repeated measures ANCOVA excluding those who reported a lower mean than the baseline mean of 2 (*n* = 262), we still uncovered no significant effects of the interaction between time and being in the intervention group [Wilks’ λ= 1, *F*(1,148) = 0.00, *p* = 0.952, η^2^ = 0.000]. This was also true when testing just secondary tasks [Wilks’ λ= 0.995, *F*(1,148) = 0.70, *p* = 0.403, η^2^ = 0.005] and both mobile phone categories [Wilks’ λ= 0.959, *F*(1,148) = 0.76, *p* = 0.386, η^2^ = 0.005].

#### Testing Plans Directly

Testing effects on a high level of abstraction, operating with means and groups, may cause some diffusion of intervention effects. To explore the behaviors that respondents actually had planned to reduce, we ran further repeated measure ANCOVAs with simple contrasts.

First, we chose the four behaviors with the highest reported baseline occurrence (i.e., “Operating the radio,” “Prolonged eye-contact with passenger,” “Reaching for an object in the car,” and “Answering incoming calls”). Thereafter, we coded a new variable to be able to test the effect of general intervention against controls, and to test specific planners from the intervention against controls. Results suggest that those planning to operate the radio less, were successful in doing so given an alpha level of 0.05 (*n* = 123, *p* = 0.018), while the other three were not. Because of the explorative nature, a Bonferroni correction was applied to adjust alpha-levels for multiple comparisons of the four contrasts (0.05/4 = 0.013). This meant that the effect was marginally significant. The marginal results on “Operating the radio” was followed up with further tests of the effect in a hierarchical regression. Here, we put “Operating the radio” at T1 as dependent variable, and the score at T0 as independent along with the mean score of intentions and conscientiousness, and a dummy coded variable for condition in a second block. This way, one can look at the change over time while controlling for the covariates, and see if any effects change when specific planning and control conditions are introduced. No significant effect or change was found, and we were unable to reject the null hypothesis.

### Hands-Free, Actual Behavior, and Relevant Plans

Additional items measuring the degree to which people already used technical solutions to deal with their mobile phones while driving was investigated. Of the 414 included in the post-survey (T1), 161 reported using hands-free solutions and 69 put their phones on speaker. Another 166 answered using Bluetooth connections, and only 43 usually hold their phone to their ear with their hand. Seventy-four reported never picking up the phone while driving, and 45 reported using voice control. Overall, this suggests that few actually use their phone the way our plans were designed to counteract. When asking for actual behavior instead of how often they were distracted, they reported similar behavior as they had distractions. This suggests that there is little discrepancy between what people report as distractors and which possibly distracting behaviors they engage with. Questions regarding if respondents felt that the plans they had made had been relevant for them yielded encouraging results. Here, they were asked to rate the relevance of their plans to deal with the two categories of distractions on a seven-point scale, with the first end-point being labeled “to a very little degree” and the seventh “to a very large degree.” The mean for these two items were 4.12, suggesting that most people found their plans relevant. 4.52 was noted for the item “I have increased my awareness for safe driving,” and 4.94 for “I think this kind of planning could help others becoming safer drivers.”

## Discussion

We have investigated baseline levels of distractions and its predictors, and tested the efficacy of implementation intentions in reducing drivers’ distractions using two samples. In the first sample there were several novel baseline descriptions and differences. The most prominent predictors of distractions were gender, how often driven, the TPB, and extraversion and neuroticism, but not conscientiousness. In the second sample, an intervention to reduce distractions was also tested. Here, we found no effect of the intervention but one, marginally significant effect on the most occurring distractor (i.e., “Operating the radio”), and only for those who planned for that behavior specifically. There was, however, an overall decline in distracted behavior, perhaps suggesting that the study itself decreases level of distracted driving. Crucially, some of the baseline predictions were replicated in the second sample, where gender, how often driven, attitudes, and PBC significantly predicted and shared a linear relation with distractions. However, some differences appeared that could be due to the different samples, for example the absence of an effect of personality or social norms in the second sample.

### Cross-Sectional Differences and Similarities

This article has investigated similar items in different populations. This approach facilitates some comparison between the studies in addition to the description of each sample.

#### Demographic Differences (H1)

Gender was effective in predicting both categories of distractions in the first sample, and predicted overall and mobile phone distractions in the second. Age also showed a strong impact when its variance increased in the second sample. This resonates well with most previous research on young adults in general, and in a traffic context in particular (Arnett, 1996; Rundmo and Iversen, 2004; Statistisk Sentralbyrå, 2016). Young males are more accident prone, and this is largely explained by them engaging in risk-taking behavior, such as distracted driving, to a larger extent (Turner and McClure, 2003). However, others have found that females are more prone to distracted driving, which is not supported in our data (Bone and Mowen, 2006). Other included demographic variables seem to play less of a role in predicting baseline differences in distracted behavior.

How often participants had driven was a significant predictor for all categories of distractions, partially confirming the first hypothesis of the project that those driving more and farther will be more exposed to situations in which distractions could occur. This can be linked to intention viability, where a goal state only can be achieved if the prerequisite situation is encountered. If one does not drive, one cannot become distracted while driving. In addition, those who drive regularly are more likely to be comfortable driving a car, and may find the task dull at times (Bone and Mowen, 2006). Thus, they may be seeking other stimulants and become more distracted drivers.

Additionally, and based on several notes from open-comments sections in the studies, some questions regarded how relevant plans had been and whether or not they used some sort of hands-free with their mobile phones. A majority of respondents in the second sample felt that their plans were more relevant than not, that others could benefit from such an intervention, and that they had increased awareness of safety in traffic. This is encouraging and could help explain the overall decline in distractive behavior from T0 to T1. When asking about remedies for using mobile phones in cars, a minority reported holding their phone to their ear. Bluetooth solutions and hands-free were common, and may have made some of the volitional help sheet redundant, thus undermining intervention efficacy. The added range of behavioral solutions to reduce distractions from mobile phones could be addressed by future research.

#### The Theory of Planned Behavior (H2)

The second hypothesis was partially confirmed. Attitudes toward distractions, while mostly significant in both samples, seem to play more of a role in predicting differences between baseline variation in distracted behavior for a sample of high-school students. It could be inferred that young people take others’ opinions more into account, and that more negative perceived norms work as a stronger deterrent for them. Senior drivers from the general population may operate by habit to a larger extent, thus making others’ evaluations of a behavior less relevant. Some research has suggested that social norms and modeling from peers are important influences on behavior in adolescents (Beck and Treiman, 1996). Others have found that internalizing social norms is an important marker of entering adulthood (Arnett, 2001). This may indicate that interventions could target social norms among young, but rather habitual behavior for more experienced drivers. Another nuancing finding comes from investigating the two measures of PBC. Here, PBC for drivers’ distractions showed a significant impact on self-reported distractive behavior, while PBC for general driving did not. PBC is closely linked with *self-efficacy*, and has been seen as one of the variables of the TPB that can predict behavior on its own (Bandura, 1977; Ajzen, 1985). We also tested whether the effects of attitudes and PBC on behavior in the second sample were mediated by intentions, as TPB would suggest. While the best test strategy would be structural equation modeling, we sought a tentative answer to this question using the Baron and Kenny (1986) approach. Specifically, by following the four steps of their mediation analysis, we find that for distractions, it seems that intentions only partially mediate the direct effects of attitudes and PBC, while the effect of subjective norms on distractions seems to be only indirect via intentions.

#### Personality and Prediction (H3)

The third hypothesis was partially confirmed. Personality traits only showed significant predictions in the first sample, where more neurotic or extraverted respondents reported being more distracted drivers. For extraversion, this effect was only present for mobile phone use and not for secondary tasks, which may suggest that the social aspect of a phone draws these people to expose themselves to such risks. Social attention has been argued as a cornerstone of the extraversion trait, and this desire to be relevant to other people may cause them to get distracted while driving (Ashton et al., 2002). Furthermore, extraversion has been linked to reckless behavior and sensation-seeking, which could further increase the trait’s predictive value in this context (Arnett, 1996; Rundmo and Iversen, 2004). These facets seem to combine to predict phone distractions, but not the inherently less social secondary task distractions.

Neuroticism significantly predicted distractions in both categories in the first sample. Two different mechanisms may be at play for each of the categories of distractions. First, these respondents may exhibit a proneness to reactive distraction, and mobile phone distractions can be the result of a bottom-up reaction to a stimulus (Jovanović et al., 2011). Secondly, some suggest that both high and low scores of neuroticism is bad for traffic safety; a high score may yield mood deviations and instability, making drivers obsess over details, while a low score may yield a lack of concern or too much reliance on other traffic safety measures (Lajunen, 2001). A neurotic driver may thus be more affected by things that are “off” when driving, so as to increase reported distractions.

In both samples, there was a lack of predictive efficacy from conscientiousness. Other research has produced contradictory results regarding conscientiousness and drivers’ behavior (Bone and Mowen, 2006; Dahlen and White, 2006). Some have noted the conflicting evidence regarding conscientiousness and accidents or reckless behavior while arguing for its efficacy (Clarke and Robertson, 2005). A generally conflicting field of evidence could result from the application of the trait to somewhat different domains. Applications to health-related behavior (Bogg and Roberts, 2004), traffic crashes (Arthur and Doverspike, 2001), accident risk (Sümer et al., 2005), and generally deviant behavior (Salgado, 2002), have, however, yielded significant effects. These results suggest that distractions in traffic, different to accidents, are an area with less impact of conscientiousness. These results warrant further investigation.

### Intervention Results (H4, H5, H6)

#### Learning from an Ineffectual Intervention

Overall, the effect of the implementation intention intervention was less than expected; hypotheses four, five, and six were all rejected. This also means that my hypothesized mediators of planning effects lack a proper test, as we only uncovered one marginally significant effect. One possible reason for the failure to find an intervention effect is lack of engagement. There are several links between respondents’ intolerance to alternatives, cognitive simplicity and how they respond (Knowles and Nathan, 1997). This highlights that acquiescence responding can be a problem in addition to social responding or desirability bias. A questionnaire that asks too much of respondents’ capacity, beyond their ability or interest, can thus directly impact how they answer and whether they engage with the study. In this case, the mental load asked of the participants may have been too large, including several demographic and personality variables, measures of the TPB, baseline behavior, in addition to the eventual forming of plans. Without much incentive, many respondents may have failed to engage properly.

It is proposed that just reading about critical situations will engage the reader in simulation (Martiny-Huenger et al., 2015). No conscious intent is supposed to be needed for behavior change (Bayer et al., 2009). Yet, evidence suggests that forming booster plans increases the impact of these interventions, suggesting that more engagement may increase its efficacy (Chapman and Armitage, 2010). This may be especially true when using a volitional help sheet where participants do not write their own plans. Because lack of engagement is an issue, an easy-to-use and entertaining technical module may mitigate this. Interactivity is suggested as a key feature of internet-based intervention designs, and that engaging designs lead to better retention (Hurling et al., 2006). Digital designs of this kind are a relatively novel and rapidly growing field of systematic study, and this research adds some to the existing knowledge by pointing at weaknesses that may undermine effectiveness (Portnoy et al., 2008).

#### Operationalization and Effect Diffusion

The operationalization of distractive behavior may be further improved by future research. Not just in terms of the structure of the 11 chosen behaviors, and their belonging to certain categories, but their relevance to actual distractions. For example, while many may become distracted by mobile phones, it is evident that several systems are in place to mitigate this. A range of new possible plans spawn when introducing these technical systems. A Bluetooth connection that does not work properly can be as distracting as a phone call, but needs an entirely different plan to reduce distractions in traffic. A solution where each respondent chooses their own baseline behaviors and distractors, and thus plans for these distractors specifically may increase intervention efficacy. This gives increased relevance for each participant, in addition to making each distractor easier to follow-up. As suggested by the one significant effect, a diffusion of actual planning effects on specific behaviors can drown in noise if the behaviors are compiled into a single index of distractive behavior. Furthermore, statistical noise could arise from respondents answering that they never were distracted by most items, and very often by a single factor. A driver may have most distractors under control, but still often get distracted by the radio or if someone calls. These individual variations would also have gotten lost in the averaging of behaviors across participants, and would require further exploratory analysis to disentangle.

#### A Question Behavior Effect?

There was an overall decline in distracted behavior from T0 to T1. This decline was not accounted for by the intervention, and it seems it was due to the passing of time alone. It can be argued that the surveys themselves may have had some impact. Because of the design of the study, this could not be tested. It could be argued that those with intentions to be safe drivers only needed a reminder and a push that was the baseline questions in order to become safer drivers. By knowing that the study inquired about distractive behavior, and that they would be measured at multiple time points, they may have been extra conscious of their behavior and exhibit a question behavior effect (Morwitz and Fitzsimons, 2004). It is proposed that being measured on one’s intentions specifically may increase the availability of specific thoughts and attitudes about a certain behavior. The results can also be the result of a Hawthorne effect, where people can alter behavior when they think they are being subjected to an effective intervention (McCarney et al., 2007). Therefore, further research should include either less engaging control groups or both an active and passive control group. Wording of information and questions should also be kept as neutral, yet engaging, as possible.

### Limitations

While biased in certain ways, self-report data still hold some validity. In many contexts including this, it is an easy and efficient access to many data points. Self-report data has also been shown to correlate highly with observed behavior in similar domains (Elliott et al., 2007). However, social desirability (Podsakoff et al., 2003), order effects (Hogarth and Einhorn, 1992), such as primacy or recency, or a range of other response biases may be at play (Furnham, 1986). Still, this is a convenient and efficient way of gathering satisfactory data, and steps were taken to reduce these biases. A passive control group could also be included to filter out potential question behavior effects and to examine the degree to which this exists in this domain. Several post-surveys could also gauge the long-term effects of such an intervention.

This project contained a number of hypotheses, and many different tests. The article tries to explore new territory and therefore contains many variables. However, there is an important disjuncture between simple exploration for the sake of finding novelties and the testing of hypotheses. The recent crisis in social psychology suggests that fishing for significant results, along with few participants and lack of replication lead to unreliable science (Earp and Trafimow, 2015). Researcher degrees of freedom refer to how researchers can change their goals and analytic methods *ad hoc* and only report statistically significant effects. Therefore, it is important to consider adjusting alpha levels (for example using Bonferroni corrections), and being clear when one tests specific, pre-determined hypotheses and when one goes exploring for interesting effects and relationships. We have tried to be clear about these issues, but there still may be reason to argue that a tighter experimental control and more stringent alpha levels should be exercised. As previously discussed, some items and scales would also benefit from re-operationalization to better fit a normal distribution. For analyses and statistical inferences, however, assumptions were met. We tested for homoscedasticity and collinearity, as well as normally distributed residuals.

### Societal Implications and Future Research

This article provided novel tests of individual differences in distractive behavior, both in a sample of high school students and a more general sample, and a description of distractions. While the interventions largely failed to prove their utility, further research should focus on making the digital volitional help sheets better, as this possibly could be a an affordable way of delivering an effectual intervention to drivers to great benefit. Furthermore, tests of baseline differences suggest that there are systematic differences between who gets more distracted. For example, results indicate that behavior change could target social factors among young, but rather on habitual behavior for more experienced drivers. Younger males with bad attitudes and poor PBC seem to generally be a group associated with greater risk, which should be focused on for future campaigns. This should also be considered when designing future volitional help sheets in this context.

Future research could include measures of sensation-seeking, risk-taking, and related factors to investigate whether these impact distractions directly. Additionally, a re-operationalization of distractive behavior is recommended, where diffusion of effects by averaging items into a mean or an index is considered.

## Conclusion

In this article, results from two different samples were reported. The purpose was to investigate the nature of distractions in traffic, test predictors of baseline levels of distractions, and to test the efficacy of implementation intentions in reducing distractions. In the first sample, only baseline data was considered, while the second also tested an intervention. In both cases, people did not generally report being very distracted. The TPB turned out as a significant predictor of baseline distraction, along with age and gender and how often respondents had driven. Personality significantly predicted distractive behavior in the first sample, but not in the second. The intervention in the second sample largely failed to provide significant reduction of distractions, but there was an overall decline. Marginal effects on the most occurring distractor for those who planned for that specific distractor, suggest that one would benefit from studying single behaviors instead of means. Societal implications and possible improvements for future research and application are discussed. To maximize the effect of internet-based interventions an engaging digital design focusing on specific behaviors and plans may important.

## Ethics Statement

This study was carried out in accordance with the recommendations of Norwegian Centre for Research Data with written informed consent from all subjects. All subjects gave written informed consent in accordance with the Declaration of Helsinki. The protocol was approved by the NSD.

## Author Contributions

All authors listed have made a substantial, direct and intellectual contribution to the work, and approved it for publication.

## Conflict of Interest Statement

The authors declare that the research was conducted in the absence of any commercial or financial relationships that could be construed as a potential conflict of interest. The reviewer SL and handling Editor declared their shared affiliation.
