# Supplementary material for: “Maybe I Will Just Send a Quick Text…” – An Examination of Drivers’ Distractions, Causes, and Potential Interventions
Source: Front Psychol. 2017 Nov 17;8:1957. doi: 10.3389/fpsyg.2017.01957 (PMC5698797; doi:10.3389/fpsyg.2017.01957)
Supplement: Supplementary file 1 [file Data_Sheet_1.DOCX]

Supplementary Material

Article Title

“Maybe I Will Just Send a Quick Text…” - An Examination of Drivers' Distractions, Causes, and Potential Interventions

Ole J. Johansson^1*,^ Aslak Fyhri^1^

^1^Institute of Transport Economics, Oslo, Norway.

*** Correspondence:**Ole J. Johansson
[ojj@toi.no](mailto:ojj@toi.no)

# Supplementary Figures


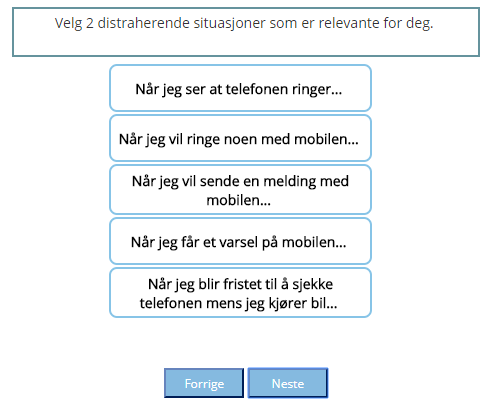


**Supplementary Figure 1.** The first screen of the digital volitional help sheet. Here respondents are asked to choose two relevant distracting situations.


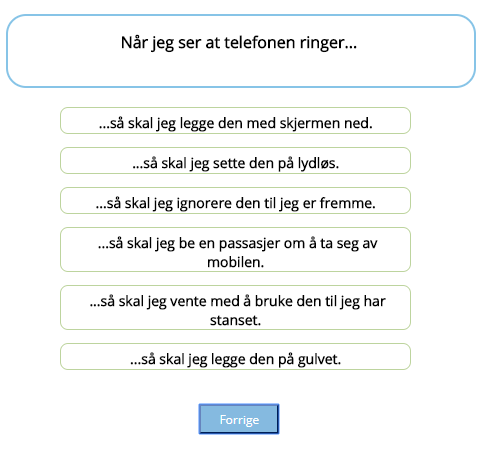


**Supplementary Figure 2.** The second screen of the digital volitional help sheet. Here respondents are asked to solve their problems with pre-defined solutions by dragging it to the problem.


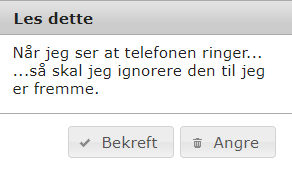


**Supplementary Figure 3.** The third screen of the digital volitional help sheet. Here respondents are asked to confirm their implementation intention plan.


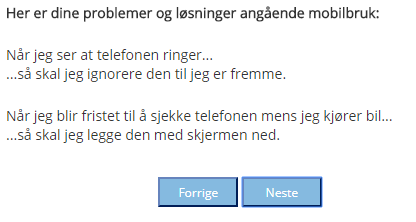


**Supplementary Figure 4.** The fourth and final screen of the digital volitional help sheet. Here respondents are presented with their two plans for mobile phone use. This four-step process is repeated as many times as desired.
